# CMEIAS-Aided Microscopy of the Spatial Ecology of Individual Bacterial Interactions Involving Cell-to-Cell Communication within Biofilms

**DOI:** 10.3390/s120607047

**Published:** 2012-05-29

**Authors:** Frank B. Dazzo

**Affiliations:** Department of Microbiology and Molecular Genetics, Michigan State University, East Lansing, MI 48824, USA; E-Mail: dazzo@msu.edu; Tel.: +1-517-884-5394

**Keywords:** bacterial cell-to-cell communication, biofilm, calling distance, CMEIAS, colonization behavior, ecophysiology, geostatistics, image analysis, spatial pattern analysis

## Abstract

This paper describes how the quantitative analytical tools of CMEIAS image analysis software can be used to investigate *in situ* microbial interactions involving cell-to-cell communication within biofilms. Various spatial pattern analyses applied to the data extracted from the 2-dimensional coordinate positioning of individual bacterial cells at single-cell resolution indicate that microbial colonization within natural biofilms is not a spatially random process, but rather involves strong positive interactions between communicating cells that influence their neighbors' aggregated colonization behavior. Geostatistical analysis of the data provide statistically defendable estimates of the micrometer scale and interpolation maps of the spatial heterogeneity and local intensity at which these microbial interactions autocorrelate with their spatial patterns of distribution. Including *in situ* image analysis in cell communication studies fills an important gap in understanding the spatially dependent microbial ecophysiology that governs the intensity of biofilm colonization and its unique architecture.

## 1. Introduction

Our team of microbiologists, mathematicians and computer scientists has been developing a suite of software applications for computer-assisted microscopy to enhance studies of microbial ecophysiology in natural and managed habitats. The long-range goal is to build a computing toolkit that strengthens microscopy-based approaches for understanding microbial ecology at spatial scales directly relevant to their ecological niches, without the need for cultivation [1]. The software suite is named **CMEIAS** (for **C**enter for **M**icrobial **E**cology **I**mage **A**nalysis **S**ystem), and as components become fully developed and documented, they are released for free download at the project website [1]. The CMEIAS ver. 3.10 upgrade currently under construction [2] includes a spatial ecology module, based on motivation to create quantitative tools that will help users obtain better, statistically defendable answers to pertinent questions of microbial ecophysiology that are influenced by spatial patterns of microbial distribution during biofilm colonization on biological and non-biological surfaces.

A central goal in spatial ecology is to define what a measured characteristic at one location can reveal about that same variable at neighboring locations. Analyses of *in situ* spatial ecology are designed to scrutinize patterns of distribution at a given spatial scale and produce predictive models of microbial colonization behavior that can help to reveal ecophysiological processes on surface habitats. Of central importance in that assessment are tests for patterns of complete spatial randomness. The essence of the statistical pattern analysis is to distinguish between spatial distributions of the organisms that can be explained by chance *versus* those that cannot. Complete spatial randomness implies that no microbial interactions affect the events resulting in their patterns of distribution. In contrast, significant deviations from complete randomness in spatial patterns indicate that regionalized microbial interactions between neighboring cells have affected their colonization behavior. Aggregated (clustered) spatial patterns of microbial colonization imply positive interactions that promote growth physiology among neighboring cells, whereas uniform patterns of distribution imply negative (inhibitory) microbial interactions resulting in their maximal, over dispersed, self-avoiding colonization behavior. This information is of significant ecological importance because spatial heterogeneity resulting from both types of nonrandom interactions between individuals tends to stabilize ecological systems [3,4]. Also, knowing the location and intensity of clustered behavior for organisms can improve the ability to understand the underlying processes that generate and sustain the interdependent microbe-environment relationships within biofilm architectures.

At the core of these positive (cooperative) and negative (conflict) interactions that reduce spatial randomness are various types of microbial cell-to-cell communication events that regulate genes affecting their colonization behavior. The key connection between biofilm spatial ecology and cell-to-cell communication occurs when positive or negative interactions are found to be spatially autocorrelated, *i.e.*, structured to operate at spatial scales that extend sufficiently to affect neighboring attached cells. Other papers in this special issue of *Sensors* describe the biochemical and genetic details of bacterial cell-cell communication. This paper describes 18 different experimental tests on data derived from CMEIAS computer-aided microscopy to analyze the spatial ecology of bacterial colonization within biofilms, providing (in the broadest sense) both indirect and direct evidence of bacterial cell-to-cell communications mediated by environmental sensing phenomena and the geospatial scale at which they occur *in situ* at individual, single-cell resolution.

## 2. Experimental Section

### 2.1. Indirect Examination of Bacterial Cell-to-Cell Communication Using Spatial Pattern Analysis

This first experimental system involved many types of spatial pattern analyses to test for microbial interactions indicative of cell-to-cell communication among individual microbes in natural biofilms. Microbial assemblages were allowed to develop on clean glass microscope slides submerged for four summer days in the Red Cedar River on the campus of Michigan State University (East Lansing, MI, USA). Slides were retrieved and their underside wiped clean. The top surfaces of the slides were mounted in water with a cover slip and examined by phase-contrast light microscopy using a 100X Planapochromat Phase 3 objective lens to resolve individual bacterial cells. Digital 8-bit grayscale images of the biofilms were acquired using a monochrome digital camera, then segmented to binary, spatially calibrated and analyzed using CMEIAS image analysis software [1,2,5,6] to produce the 2-dimensional coordinate systems that accurately define the geospatial location of individual attached bacterial cells. Extracted data were analyzed statistically using StatistiXL [7], EcoStat [8], ProStat [9], GS+ Geostatistics [10], and in-house CMEIAS Data ToolPack software [2].

The accuracy of CMEIAS image analysis software to measure and compute the object centroid-to-centroid 1^st^ and 2^nd^ nearest neighbor distances was evaluated using a high-resolution 3-frequency grid distortion target (Edmund Optics, Barington, New Jersey, USA) as ground truth. The accuracy of CMEIAS color segmentation software used to process the color images for analysis was previously measured as 99+% [11].

### 2.2. Direct Examination of Bacterial Cell-to-Cell Communication Using Rfp-Source and Gfp-Sensor Reporter Strains

The second experimental system was designed to further advance our understanding of bacterial cell-to-cell communication during their colonization of plant roots. CMEIAS image analysis was used to reevaluate the spatial scale of calling distances and the variations in intensity of gene expression activated by extracellular signal communication molecules produced by neighboring cells of *Pseudomonas putida* reporter strains during their colonization of tomato roots. Details on construction of fluorescent reporter derivatives of *P. putida* IsoF serving as the source and sensor strains for these bacterial cell communication studies using N-acylhomoserine lactone (AHL) signal molecules are previously described [12–16]. Briefly, the “AHL-source” strain produces N-hexanoyl-, N-decanoyl-, and N-dodecanoyl-acylhomoserine lactone signal molecules and harbors a plasmid with constitutive expression of the red fluorescent protein. The “AHL-sensor” strain, *P. putida* F117 (pKR-C12) has a mutation in its single copy of the *ppuI*-gene that abolishes its ability to synthesize AHLs, but contains an AHL-inducible reporter plasmid pKR-C12 with a green fluorescent protein (GFP)-encoding sensor cassette. This sensor cassette contains an AHL-regulated promoter for *ppuA* (encoding acetyl-CoA ligase) coupled to the Gfp gene that responds with high specificity and differential sensitivity to defined AHLs (C10- and C12-HSLs and 3-oxo-C10 to 3-oxo-C14-HSLs) at threshold concentrations as little as 10 nmol/L [14].

Axenically grown seedling roots were inoculated with both the AHL-producer and AHL-sensor strains of *P. putida* at 10^9^ cells per plant, grown gnotobiotically, harvested and examined by laser scanning microscopy in the epifluorescence confocal mode [15]. Georeferenced, confocal RGB images were merged into loss-less montages, segmented using CMEIAS Color Segmentation software [11], and analyzed using CMEIAS image analysis software [2,5,6] to evaluate the *in situ* spatial relationships and luminosity of segmented red-fluorescent AHL-source and green-fluorescent AHL-sensor cells colonized on the root surface. To avoid errors in measurement of *in situ* calling distances and gene expression, communicating cells attached to root hairs and cells located above or below the optisections used to prepare the montage of X | Y projected images were excluded from analysis, as was done in our previous study [15].

## 3. Results and Discussion

### 3.1. Spatial Pattern Analysis Reveals Aggregated Colonization Behavior Indicative of Bacterial Cell-to-Cell Communication

**Figure 1.**
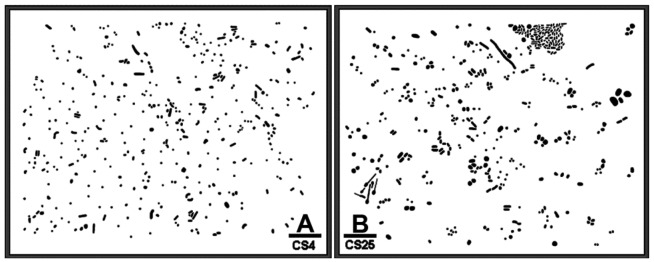
*In situ* spatial distribution of freshwater microbial assemblages in biofilms labeled as (**A**) CS4; and (**B**) CS25 on microscope slides. Bar scales are 10 μm.

Figure 1(A,B) is binary images of two natural microbial assemblages developing in freshwater biofilms on glass slides that are used here to illustrate a CMEIAS spatial pattern analysis that reveals their colonization behavior and intensity of bacterial cell-to-cell communication. Various quantitative features were extracted from each individual cell to test for complete spatial randomness in their distribution using point pattern and geostatistical methods. These spatial features included the X and Y Cartesian coordinates of each foreground object's centroid (georeferenced to the 0,0 X,Y landmark origin assigned to the image's lower left corner), the μm distance from each object centroid to its 1^st^ and 2^nd^ nearest neighboring cell, the cumulative Empirical Distribution Function of the 1^st^ nearest neighbor distance, and the CMEIAS Cluster Index that measures the clustering intensity of each cell in relation to its local environment [2,6,17]. The measured error rates of the centroid-to-centroid 1^st^ and 2^nd^ nearest neighbor distances used to compute these spatial attributes were 3.2% and 2.3%, respectively, with an overall combined accuracy of 97.2% (n = 38).

Statistical analyses indicated that the frequency distribution of the 1^st^ and 2^nd^ nearest neighbor distances and cluster indices of cells in these biofilms were significantly skewed and lacked normality (Table 1). The Mann-Whitney non-parametric test indicated that their median values were significantly different, and the cluster index was significantly more intense in the CS25 biofilm (Table 1).

**Figure 2.**
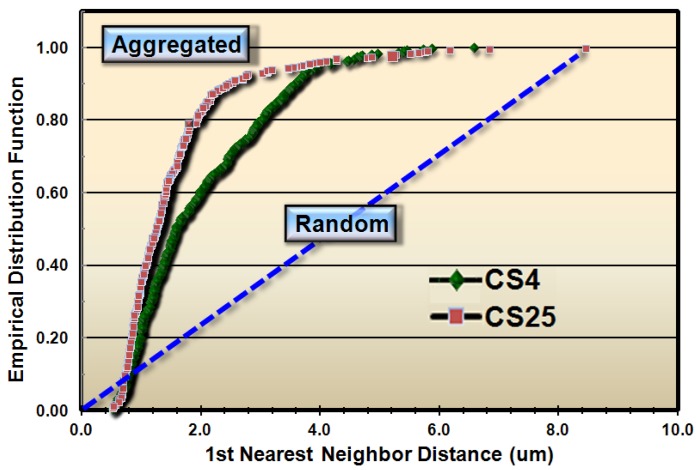
Cumulative empirical distribution function of the nearest neighbor distances between individual bacteria within images of biofilms CS4 (green diamonds) and CS25 (red squares). Differences in intensity of aggregated patterns are indicated by empirical distribution values that ascend above the diagonal dashed line of complete spatial randomness.

These results justified subsequent analyses to test whether the spatial patterns of microbial distribution deviated from complete spatial randomness, and if so, the intensity of their clustered aggregation. Several methods of spatial statistics were performed on the CMEIAS data to test this hypothesis. The first evaluation, called the *Empirical Distribution Function*, is a plot of the cumulative ranking of the 1^st^ nearest neighbor distances measured between individual cells in the sample compared to the distribution that would result if the pattern were completely random. In this latter case, the data points would distribute along the diagonal, dashed blue staircase. The resultant plot of the empirical distribution function indicated that both biofilm samples had aggregated patterns of distribution, and that this colonization behavior was more intense in biofilm CS25 (Figure 2).

The same data on 1^st^ and 2^nd^ nearest neighbor distances were evaluated by four other spatial point pattern statistical tests (Holgate Aggregation, Russ Randomness, Clark and Evans Dispersion/Spatial Density, and Hopkins and Skellam Aggregation) for complete randomness in spatial distribution of cells. These 4 tests indicate spatially aggregated patterns when the corresponding upper class limits of their indices computed from the 1^st^ and 2^nd^ nearest neighbor distances are >0.5, <1.0, <1.0 and >1.0, respectively. The results of all of these point pattern tests rejected the null hypothesis of spatial randomness in favor of the alternative hypothesis of aggregated distributions, and the intensity of this colonization behavior was significantly higher in the CS25 biofilm sample (Table 2).

Concepts derived from fractal theory are fundamental to an understanding of the landscape complexity of scale-related phenomena in ecology [18]. Values greater than 1.000 for this self-similarity statistic are indicative of the complexity of an aggregated pattern of distribution resulting from the scale-dependent heterogeneous fractal variability in limiting resource partitioning, and reflect the high efficiency at which cells position themselves spatially and physiologically when faced with the interactive forces of microbial coexistence to optimize their allocation of nutrient resources on a local scale [19]. Thus, the fractal geometry of landscapes can reflect microhabitat fragmentation and heterogeneity in resource utilization rates [19]. CMEIAS fractal dimension analysis of Figure 1(A,B) indicated that the bacterial distribution in both biofilm assemblages exhibited positive fractal geometry, and that the intensity and complexity of this spatially dependent adaptation was again stronger for the CS25 biofilm (Table 2).

The cluster index, computed as the inverse of 1st nearest neighbor distances, is a sensitive, local *sensor* of positive (cooperative) bacterial cell-to-cell interactions affecting their colonization behavior over the entire spatial domain. High values of this metric extracted from surface-attached cells indicate that they are arranged in spatially aggregated patterns that facilitate cell communications resulting in positive metabolic cooperations promoting their localized growth into populations of microcolony biofilms [6,17,20].

**Figure 3.**
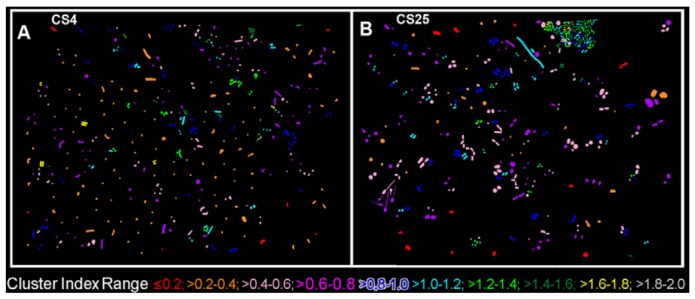
*In situ* classification of each microbial cell within the (**A**) CS4; and (**B**) CS25 biofilm landscapes into bins defined by the upper limit of their CMEIAS Cluster Index. The pseudocolor assignment for each bin class covering the range of the CMEIAS Cluster Index is indicated at the bottom of the images.

The CMEIAS 1-dimensional classifier was used to sort individual cells in the two biofilm images into bins based on division of a scale defined by a single measurement feature [5]. Figure 3(A,B) shows the classifier output of rendered images that provide a quick, visual appraisal of the binned clustered index assigned to each individual bacterium *in situ* in the CS4 and CS25 biofilms. This pseudocolored classification scheme indicates a preponderance of well-dispersed cells and a few small clusters (cluster indices < 0.4) in the CS4 biofilm (Figure 3(A)), and a larger proportion of cells grouped into aggregates (cluster indices > 0.4) in the CS25 biofilm (Figure 3(B)), (also see Table 1 median test).

**Figure 4.**
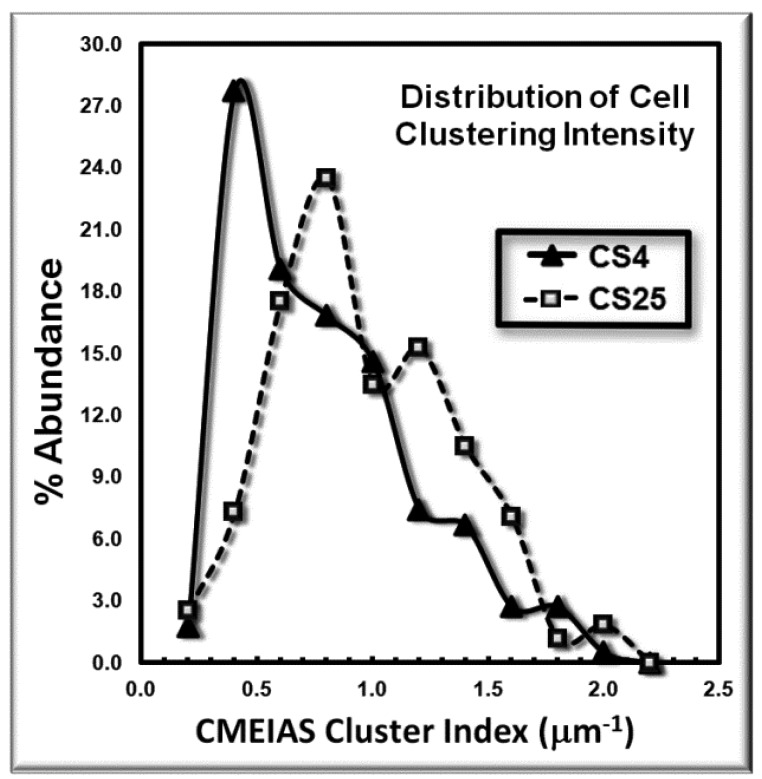
Frequency distribution of each cell's Cluster Index within CS4 and CS25 biofilms.

The frequency distribution of the cluster indices for these microbes is presented in Figure 4. This histogram plot indicates the shift to higher cluster indices for the bacteria in the CS25 biofilm landscape, indicative of their higher intensity of aggregated colonization behavior *in situ*.

**Figure 5.**
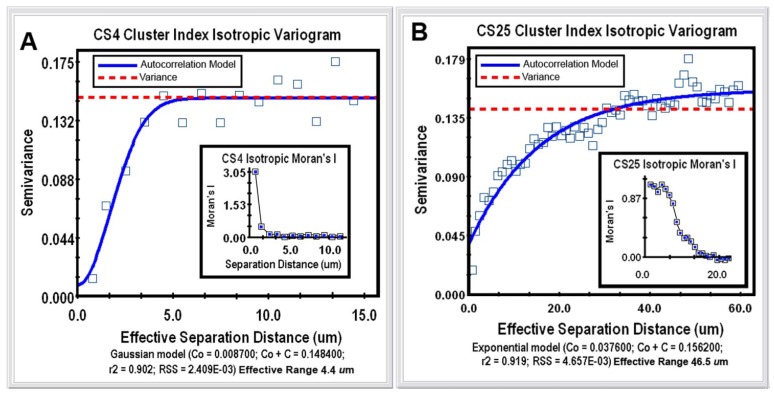
Isotropic semivariograms of the spatially autocorrelated cluster indices of cells in the CS4 (**A**) and CS25 (**B**) biofilm assemblages. Note the greater effective separation distance (95% of the asymptote) and Moran's Index (inserted plots) for cells in the CS25 biofilm, indicating that they have a stronger autocorrelated intensity of clustered distribution pattern and are positively interacting over a wider radial *in situ* spatial scale.

Geostatistical analysis is the most powerful category of spatial pattern analysis that can be used to unravel the spatial uncertainty of interactions between individual microorganisms. This geospatial method examines the continuity or continuous variation of spatial patterns over the entire domain by testing whether a user-defined, continuously distributed regionalized variable (called the “Z-variate”) is spatial autocorrelated, *i.e.*, exhibits spatially-dependent structure [21]. The analysis indicates whether cells at one location (georeferenced by its X,Y Cartesian coordinates) express the Z-variate with sufficient intensity to influence (thereby communicate) the same regionalized response of neighboring cells at another location. Thus, patterns displaying spatial autocorrelation indicate that operations involve a spatial process, *i.e.*, its intensity is significantly influenced by the location of neighboring cells, rather than occurs independent of the location of their neighbors. When found, the autocorrelation result can be mathematically modeled to accurately connect various spatially dependent relationships derived from regionalized variable theory, plus make optimal, statistically rigorous interpolation (kriging) maps of the local intensity of the measured Z-variate parameter within that spatial domain, based on analysis of its weighted average from sampled locations. From the microbial ecology perspective, cellular interactions that are autocorrelated with spatial patterns of microbial distribution within biofilms statistically indicate that communication events (in the broadest sense) significantly influence the *in situ* colonization behavior (cooperation *vs.* conflict) of neighboring cells over a defined scale within the spatial domain. At the core of geostatistical analysis is the variogram plot, which defines the extent to which the measured Z-variate exhibits spatial dependence between sample locations. It does so by examining how the values of the Z-variate become different as the spatial separation between the sample points increases. In this example, geostatistics using a simulation analysis of 10,000 subsamples were performed to test for connectivity of each georeferenced cell's cluster index values to their spatial patterns within the 2 biofilm image domains. Semivariance analyses of the clustered indices of the bacteria in the CS4 and CS25 biofilms indicated that this regionalized variable positively autocorrelated with the spatial pattern of distribution (Figure 5(A,B)), and Moran's Index of the same georeferenced data indicated that the intensity of this spatially autocorrelated structure was more than twice as strong for the CS25 biofilm sample (Table 1).

The mathematical model that statistically best fits the variogram's autocorrelation data (represented by the solid blue line in Figure 5(A,B)) also indicates the effective separation range (computed as the X axis intercept at 95% of the modeled asymptote height) that each cell positively influences the measured Z variate of neighboring bacteria present. This range defines the separation distance between neighboring cells in which the measured Z-variate remains spatially autocorrelated. The Z-variates of cells at sampling points separated by more distance than the maximum of the effective range are independent of one another. In this study, the effective separation range at which each cell positively influenced the aggregated colonization behavior of its nearest cell neighbors extended out to a radial distance of 46.5 μm in the CS25 biofilm sample as compared to 4.4 μm in the CS4 biofilm sample (Table 2). Thus, the *in situ* spatial scale of the separation distance at which bacterial cells exhibit positive, autocorrelated clustering behavior was more than 10-fold longer for cells in the CS25 biofilm sample (Table 2). These radial effective separation distances encapsulate 96% of the bacteria in the CS4 biofilm and 100% of the bacteria in the CS25 biofilm. These results statistically define the *in situ* spatial scale of that positive, autocorrelated cell-to-cell interaction where the bacteria have attached and the highly common occurrence at which those spatially dependent, cooperative communication events occur. The results illustrate how the powerful method of geostatistics can be used to analyze bacterial sensing of their neighbors and precisely define the real-world spatial scales at which they occur *in situ* during biofilm development.

**Figure 6.**
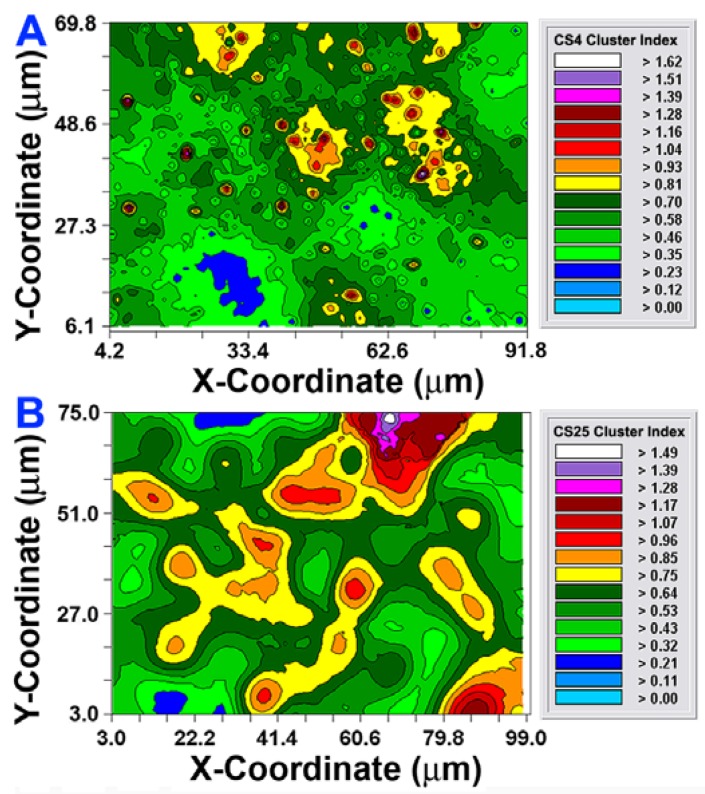
2-dimensional Kriging maps of the spatially autocorrelated local cluster indices for the microbes within the CS4 (**A**) and CS25 (**B**) biofilm samples. Note the significantly greater patch size and interpolation of higher intensity of clustered patterns in the CS25 biofilm (**B**).

After the autocorrelation model was optimally fit to the data, 1,000 simulations of multigrid refinements of the model were computed to produce the corresponding kriging maps. These high-resolution pseudocolored graphics elegantly map the spatial autocorrelation in the data. This knowledge is used to derive accurate, unbiased estimates of the spatial continuity of Z-variate values within the sampling unit, thereby precisely resolving detailed spatial patterns with known variance for each interpolated point [21]. In this study, the kriging maps depict the centers of local clustered cell intensities that provide a vivid, geostatistically autocorrelated, continuous interpolation of the spatial variability in aggregated colonization behavior over the entire spatial domain, even in areas not sampled (Figure 6(A,B)). Included in the kriging maps are isopleth lines whose curvature connects points of equal intensity (like a weather map). The pseudocolored, intensity-scaled Z-variate and isopleth-defined contours provide clear recognition of the larger patch sizes and greater intensities of this clustered colonization behavior in the CS25 biofilm sample. This experimental test result maps the similarities and differences in the spatial ecology of cells communicating their clustered colonization behavior with neighboring cells.

Considered collectively, the results presented in Figures 2–6 and Tables 1–2 provide multiple, statistically significant indications that microbial colonization within these natural biofilms is not a spatially random process, but rather is controlled by strong, positive cooperative interactions between communicating cells that influence their neighbors' aggregated colonization behavior. Statistical evaluation of the CMEIAS image analysis data extracted from images of the CS4 and CS25 biofilm assemblages at single-cell resolution indicate that the intensity and spatial scale over which these positive cell-to-cell communication events occur are consistently and statistically stronger for the CS25 biofilm sample and encapsulate all of the cells in that biofilm landscape.

### 3.2. Direct in situ Geospatial Analysis of Cell-to-Cell Communication by Individual Bacteria during Their Colonization of Plant Roots

#### 3.2.1. Analysis of *in situ* Calling Distances

It is generally thought that high bacterial population densities are needed to exceed the threshold level of AHL signal concentrations required to activate genes and their physiological functions. Hence, this specific type of microbial communication has become known as ‘quorum sensing’ that functions primarily as sensors of high population density, thus optimizing the expression of functions that are most beneficial when simultaneously performed by dense populations. Despite its wide appeal, this quorum sensing paradigm has been challenged since the methods commonly used to detect it ***require*** high populations, and neither the need for group action nor the selective conditions required for its evolution have been definitively demonstrated [22]. Recently developed technologies using fluorescent reporter strain constructions, confocal microscopy, computer-assisted digital image analysis, and geostatistical modeling for single-cell resolution experiments have been used to measure the *in situ* spatial scale and local population requirements of AHL-mediated cell-to-cell communication during bacterial colonization of plant roots [15]. These stimulus-response studies using genetically engineered reporter strains of *Pseudomonas putida* have shown that this form of cell communication can be accomplished by single, individual bacterial cells that are separated from each other and from high population densities by relative long-range distances. Rhizobacteria were able to conduct cell-to-cell communication on roots with a minimum quorum requirement of two cells (source and sensor) and a maximal *in situ* separation ‘calling distance’ of up to 78 μm (equivalent to two people talking to each other while standing approximately 130 m apart). In addition, individual bacteria in small clusters (2–3 cells) communicated with each other even when separated from dense populations by even longer distances. Thus, during colonization of plant roots, one individual bacterium is able to produce sufficient AHL signal molecules to communicate with another single bacterial cell neighbor even when those two cells are separated apart by a wide range of distances. Geostatistical modeling of local spatial densities predict that AHL-mediated cell-to-cell communication is governed more by the *in situ* spatial proximity of cells within AHL gradients than by a quorum requirement of high population density, and therefore this type of cell-to-cell interaction is likely to be more commonplace in biofilms than originally thought [15]. Follow-up studies [23,24] have tested this hypothesis in 3-dimensional analyses of other experimental model systems and confirmed that as few as two individual bacterial cells can communicate with each other over long-range distances when confined to microenvironments with low cell density. In this study, the scale of calling distances for rhizobacterial communication is addressed on another landscape of the tomato root surface to further test the hypothesis that bacteria communicate with one another via AHL signal molecules even when they have not congregated into high local population densities.

**Figure 7.**
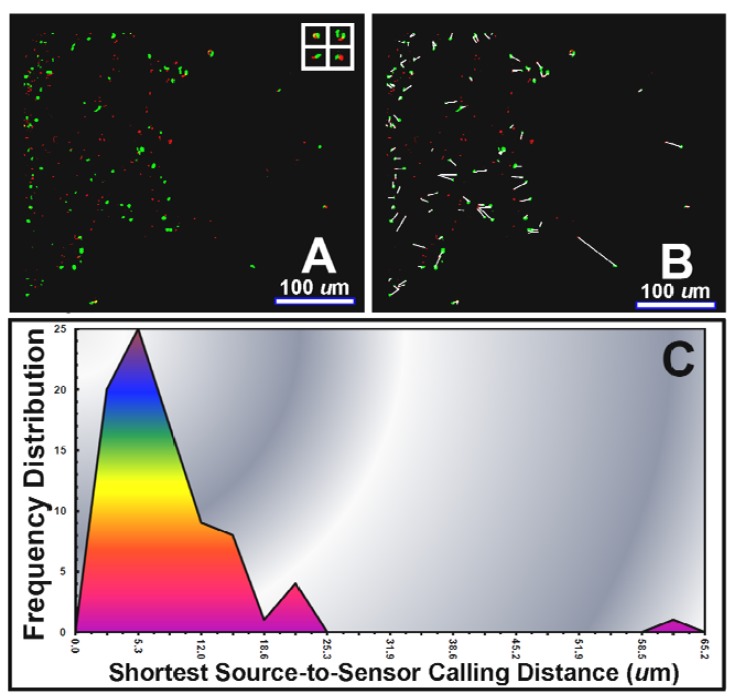
Computer-assisted microscopy of AHL-mediated cell-to-cell communication between red-fluorescent source and green-fluorescent sensor reporter strains of *Pseudomonas putida* colonized on a tomato root. (**A**) Confocal optisection image of AHL-source (red) and AHL-sensor (green) cells on the root surface. Inserted images enclosed within white frames are examples of small clusters of communicating cells located at long distances from high population densities; (**B**) CMEIAS-rendered analysis image of the separation distances between neighboring pairs of communicating cells; (**C**) Frequency distribution of shortest source-to-sensor calling distances. Bar scales equal 100 μm. Gradient graphics are included for emphasis.

An example of direct *in situ* evidence of AHL-mediated cell-to-cell communication during bacterial colonization of a tomato root is provided in Figure 7(A). In this case, the green-fluorescent pseudomonad sensor cells are positioned to perceive sufficient concentrations of the AHL signal molecules within gradients made by the red-fluorescent pseudomonads, and respond through gene expression of green fluorescent protein. CMEIAS image analysis at single pixel resolution of the shortest linear distance between each activated (green-fluorescent) sensor cell and its 1^st^ nearest neighboring (red-fluorescent) AHL-source cell (Figure 7(B)) indicated a frequency distribution of calling distances with a mode of 5–6 μm and maximum of 63 μm (Figure 7(C)). These descriptive statistics are similar to our earlier results (mode of 4–5 μm, maximum of 57–78 μm) of a larger analysis of bacterial cell-to-cell communication during colonization on tomato and wheat roots [15]. Control experiments indicated that the gnotobiotic culture systems were maintained free of extraneous microbes (including AHL-producers) and that the AHL-sensor strain did not fluoresce green when inoculated alone on the plants, thereby excluding a false-positive result due to autoinduction or activation by an AHL-mimicking compound of plant origin. Of course, the three-dimensional scale of such AHL gradients in natural rhizospheres would be influenced by the extent of its adsorption to the root and to inanimate soil particles (e.g., clay and humic particulates), quenching by AHL-degrading enzymes made by the plant or other neighboring microbes, and resistance to diffusion by the viscous, discontinuous liquid films covering the root [15].

#### 3.2.2. *In situ* Intensity of Bacterial Gene Expression Triggered by AHL Signal Molecules

**Figure 8.**
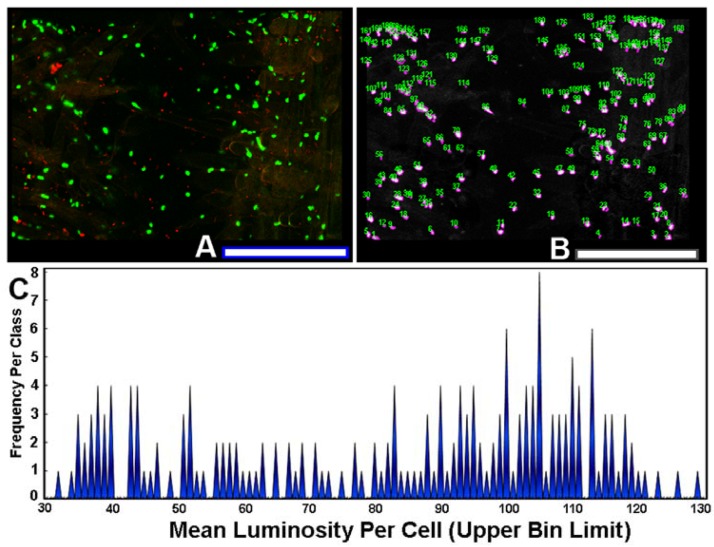
(**A**) Red fluorescent AHL-source and green fluorescent AHL-sensor strains colonized on a tomato root surface; (**B**) Color segmented and annotated cells of green fluorescent AHL-sensor cells; (**C**) Frequency distribution of the mean luminosity per green fluorescent AHL-sensor cell. Bar scales equal 100 μm.

The use of sensor cells that report intracellular fluorescent proteins permits the measurement of expression levels of gene activation by AHL gradients *in situ*. To quantify the variation in this ecophysiological communication response, each individual AHL-sensor green-fluorescent cell in the image (Figure 8(A)) was color segmented (Figure 8(B)), and its mean luminosity value was extracted by image analysis. The histogram plot of mean luminosity per cell indicates significant cell-to-cell variation in AHL-activated gene expression, with a multi-modal distribution containing at least 15 resolved bins of fluorescent brightness intensity of the intracellular green fluorescent protein (Figure 8(C)). Thus, rhizobacteria communicate with each other while they colonize root surfaces, discussing many scientific topics over a wide range of intensity levels, figuratively from a “soft whisper” to an “intense shouting.” This variation in AHL-activated autoinduction (gene expression) likely reflects, *inter alia*, their spatial position within the concentration gradients of the signal molecules at the root surface. This experimental test result illustrates the awesome power of computer-assisted microscopy to quantitatively measure bacterial cell communication at single-cell resolution, and also leads one to predict that commonly used population-level measurements of gene expression by sensor strains may grossly underestimate the real-world variation in these same signal transduction responses within biofilms.

## 4. Conclusions

Albert Einstein once said: “Sometimes what is counted doesn't count, and what really counts cannot be counted!” Studies on cell communication within microbial biofilms can benefit by incorporating a spatial context, because in this habitat, “spatial relationships really matter”. This paper describes how CMEIAS-assisted microscopy can help measure what really counts in this field: quantitative data in spatial ecology that bestow new insights to help piece together the complicated story of biofilm development by gaining a clearer understanding of spatially-dependent interactions involving cell communication at real-world spatial scales important to the microbe's perspective. Characterizing the spatial scale of bacterial interactions resulting from cell-to-cell communication is important because it is a strong determinant of spatial patterns that reflect their colonization behavior, and certain ecological processes may operate at a particular scale but not at all at a different scale.

Eighteen different experimental tests were described using two types of sensors. Sixteen tests using the first sensor type (nearest neighbor-based cluster indices) differentiated the intensity of autocorrelated spatial structure in aggregated colonization behavior between two natural freshwater biofilms. Two other tests using the second type of sensor (genetically engineered strains to report signal molecule production and perception) indicated the range of calling distances between individual bacterial cells *in situ* and the variation in intensity of that cell-to-cell communication. These quantitative methods of spatial ecology performed at single-cell resolution provide statistically definitive information on bacterial sensing events that far surpasses what can be inferred from a visual interpretation of micrographs alone. They are also more accurate and precise in defining the real-world minimum quorum requirements, intensity and spatial scale of bacterial communication than are other methods that require higher populations to detect positive signals. Applying the central principles of spatial pattern analysis to cell communication events within microbial biofilms fills an important gap in understanding the strategies and forces that govern microbial ecophysiology while colonizing surfaces leading to variations in their biofilm architecture.

Spatial ecology studies reported here confirm and expand upon previously acquired data [15,23,24] indicating that bacteria within biofilms communicate with each other at both low and high local cell densities. They indicate again that one individual cell can produce and excrete enough signal molecules allowing it to communicate with and activate genes in a neighboring cell. Cells colonized on plant root surfaces exhibit significant cell-to-cell variation in the intensity of this stimulus-response. These studies also indicate that cells within biofilms are arranged in aggregated patterns *in situ*, and predictably, that autocorrelated colonization behavior facilitates positive metabolic cooperations that promote their growth into microcolonies [6,20]. The significance of these results support the model that microcolony development can help microbes to maintain their ecological niche and gain the cooperative benefits of multi-cellularity within biofilms, that spatial aggregation can readily produce local cell densities that exceed the threshold concentration of signal molecules needed for cells to communicate with each other, and that maintaining honesty in signaling can be avoided when the signaling cells grow into microcolonies [25].

Future work should continue to further test and validate these ecophysiological models of cell communication and colonization behavior by microbes at single-cell resolution during dynamic stages of biofilm development, including their spatiotemporal redistribution within various natural habitats.

## Figures and Tables

**Table 1.** Statistical inference tests of the distributions of nearest neighbor distances and clustered indices between individual bacteria within images of the CS4 and CS25 freshwater biofilm assemblages.

**Statistical Test (accept or reject stated condition)**	**1^st^ Nearest Neighbor Distance (μm)**		**2^nd^ Nearest Neighbor Distance (μm)**		**CMEIAS Cluster Index (μm^−1^)**	

	**CS4**	**CS25**	**CS4**	**CS25**	**CS4**	**CS25**
Normal Distribution? (Shapiro-Wilks W)	0.906 ^a^ (No)	0.715 ^a^ (No)	0.937 ^a^ (No)	0.817 ^a^ (No)	0.924 ^a^ (No)	0.979 ^a^ (No)
Significant Skewness?	1.028 ^a^ (Yes)	2.794 ^a^ (Yes)	1.143 ^a^ (Yes)	1.922 ^a^ (Yes)	0.848 ^a^ (Yes)	0.399 ^a^ (Yes)
Median	1.591	1.257	2.711	1.905	0.629	0.796
Significantly Different Median? (Mann-Whitney U)	CS4 > CS25 (Yes, U = 115293 ^a^)		CS4 > CS25 (Yes, U = 113652 ^a^)		CS25 > CS4 (Yes, U = 112649 ^a^)	

a Significance level is indicated when the P value was ≤0.05.

**Table 2.** Ecological statistics to test if the microbial distribution in the CS4 and CS25 biofilms deviate from complete spatial randomness, and compare the intensity of spatial aggregation in their colonization behavior.

**Spatial Pattern Metric**	**Features Used**	**CS4 (p <)** ^b^	**CS25 (p <)** ^b^	**Cluster Intensity**
Holgate Aggregation	1^st^ & 2^nd^ NND ^a^	0.556 (0.001)	0.561 (0.001)	CS25 ≫ CS4
Russ Randomness	1^st^ & 2^nd^ NND	0.961 (0.01)	0.727 (0.001)	CS25 > CS4
Clark & Evans Dispersion	1^st^ NND, Spatial Density	0.929 (0.01)	0.764 (0.001)	CS25 > CS4
Spatial Density (cells/mm^2^)	Cell Count per Image Area Normalized to mm^2^	54,735	63,458	CS25 > CS4
Hopkins & Skellem Aggregation	Random Point to Nearest Object, 1^st^ NND	1.523 (0.000)	12.582 (0.000)	CS25 ≫ CS4
Fractal Geometry	Cumulative Intersection	1.892	2.140	CS25 > CS4
Effective Range Separation Distance	Object Centroid X,Y Coordinates, Cluster Index	4.4 μm	46.5 μm	CS25 ≫ CS4
Moran's I Spatial Autocorrelation Index	Object Centroid X,Y Coordinates, Cluster Index	4.244	10.579	CS25 > CS4

a NND = nearest neighbor distances;

b P values are indicated in parentheses.
